# Classification and Causation of Diseases

**Published:** 1877-03

**Authors:** C. B. Johnson

**Affiliations:** Tolona, Illinois


### CLASSIFICATION AND CAUSATION OF DISEASES.

By C. B. JOHNSON, M.D., Tolona, Illinois.

[Abstract of a paper read before the Champaign Co. Med. Society, Jan. 9, 1877.]

Various divisions and systems of classification of diseases have from time to time been presented by eminent medical men. To mention only some: One division is made according to duration, and by this method diseases are separated into acute and chronic. A second is by location; and here ailments are referred to as affecting this or that particular anatomical division of the body. A third system is based upon symptomatology. A fourth upon morbid anatomy; while a fifth is founded upon causation.

But notwithstanding this diversity in the modes of classification, in modern medicine, at least, the several varieties may be referred to one of three systems, viz., either the one grounded upon symptomatology, or the one upon pathological anatomy, or yet the one upon etiology.

Substantially it may be said that the symptomatological school was introduced, and in a measure perfected, by the English; the pathological by the French, and the etiological by the Germans.

About a hundred years ago Dr. Cullen, of the University of

Edinburg, gave to the world his "Nosologia Methodica," in which a systematic division of diseases was made into classes, genera, species, etc., in the same manner that plants in botany and animals in zoology are arranged.

This system was based upon symptomatology, and at the time of its introduction was by far the most acceptable method that had been proposed; and, with various modifications, was the one very generally adopted till about the close of the first quarter of the present century. According to it, diseases were known by their appearances only, and a given malady was assigned or refused a place in a certain group of diseases in accordance with the fact of its symptoms agreeing with or differing from other members of that particular group.

By those who introduced it, an effort was seemingly made to discover for each disease a peculiar symptom which should be pathognomonic of that particular ailment, and around which all the other symptoms should cluster to form a perfect whole. But notwithstanding the popularity of Cullen's classification, it was in reality exceedingly cumbersome, for it embraced over 1,300 diseases, with a different treatment for each.

In the year 1808, Brouissais, of France, published his "Chronic Phlegmasia"; this, so to speak, opened up the new science of pathological anatomy, and was followed in due time by works on the latter subject from Louis, Andral, Cruveilhier, and a host of others in France.

In the year 1820 Brouissais published another work entitled "Examinations into Medical Doctrines and Systems of Nosology," in which the assertion was made that 60 out of every 100 diseases had their origin in gastro-enteritis; that continued fever, in its beginning, was only an inflammation of the stomach and bowels; small-pox and the exanthemata generally were but cutaneous inflammations, kept up by the gastro-enteritis, and rheumatism and gout were often caused by irritation of the stomach, and could frequently be cured by local applications to the gastric region. Indeed, according to his ruling, local diseases absorbed nearly the whole category of ailments, while constitutional maladies were well nigh, if not completely, stricken from the lists. In fact, one of the advocates* of these views says "Diseases being deviations from the natural actions and structures of particular tissues are always local, never general."

Those who advanced these doctrines, though professing to have reached their conclusions from a careful study of pathological anatomy, called theirs the *physiological* school. In the medical literature of 50 years ago the word *ontologist* is of frequent occurrence. This was an epithet applied to one who was supposed to believe that disease was a distinct entity, which for a time took up its abode in the body. Indeed, all those who did not subscribe to the tenets of the so-called physiological school were rather opprobriously styled *ontologists*, much in the same way that the Mormons of our time call all the rest of the world *Gentiles.*

This brings us to the consideration of the etiological system. Two hundred years ago infusoria and spermatozoa were discovered, and as the microscope thus revealed the fact that there were living organisms in the human body — for spermatozoa were for a long time believed to be living animals — it did not take long for the imaginative minds of the medical men of that era to conceive the fact that swarms of amimalculæ entered the body and produced epidemic disease. And it is said that one writer of the seventeenth century gravely proposed that a great noise should be made by firing cannon, blowing trumpets, etc., hoping thus to drive away the vast flocks of these disease-producers that were believed to infest the air.

Of course, such a proposal was calculated to bring discredit upon the whole theory; and the idea of low organisms having any agency in the production of disease was substantially ignored till about the middle of this century. Since about 1850 it would appear that some German investigators have been patiently examining into the causation of disease. Their labor has, however, been more especially directed to the etiology of infectious diseases. And while it must be confessed that as yet the result of their investigations has been comparatively meagre, still the fact remains that their discoveries have

* Prof. Sam. Jackson, of the Univ. of Pen.

been of such a nature as to warrant the enlargement of that group of diseases known as infectious and contagious, and likewise to justify the framing of a new theory of their origin.

Agassiz, from a single fossil fish scale as a basis to work on, thought himself justified in constructing a model of the whole fish. Afterward a perfectly fossilized specimen of the species to which this fish belonged was discovered, and it was found to correspond in the most minute particulars with the model made by Agassiz. There is certainly a much less disproportion between the germ theory of the origin of infectious diseases and the well and authenticated facts upon which it is based, than there was between the complete model of Agassiz and the single fish scale which served him as a foundation to build on. Or, in other words, the relation between the true and the *probably* true would seem to be more intimate in the former case than in the latter. And as the revelations of time proved Agassiz correct, so may we hope, nay, almost predict, that the disclosures of the future will so substantiate this theory as to place it beyond controversy. Of the facts, however, relating to this whole subject, it may be said that as yet we have only the bud, and that the full-blown flower, and after it the completely developed fruit are yet to come.

But let us see as to the character of some of these facts: In the year 1848 Lebert, having had occasion to remove a cervical cyst from a child, noticed, after a little while, that the wound, which at first began to heal kindly, had suddenly become converted into a deeply corroding ulcer. And a closer examination revealed the fact that this bad result came from the presence of numerous bacteriæ.

This is a vegetable organism so minute that it is said to be barely visible under the most powerful microscope. Several varieties of bacteria are known to exist. Passing over the almost unpronounceable technical names, there are mentioned, among others, the rod-like, spherical, corkscrew-shaped bacteria, besides several unclassified varieties. Some of the species are known to be prolific disease producers.

The micrococcus or rod-like bacteria has been pretty clearly proven to be the cause of diphtheria. Ebert says, "without micrococci, *no diphtheria.*" Oertel states that any increase in their number is certain to be followed by increase of diphtheritic deposit.

In relapsing fever a peculiar organism is found infesting the body, and as it is seen in no other disease, it is fully believed by some to be the sole cause of that fever. In the case of malignant pustule, a certain organism is known to exist which is regarded as the propagator of that disease. The same, with a considerable degree of certainty, is inferred to be the case with pyemiæ and puerperal fever. Perhaps at this day no one can be found who will for a moment question the direct agency of the acarus scabiei, or itch mite, in the production of itch. Yet the very fact of its existence was for a long time a vexed question, and was only verified to a certainty 40 or 50 years ago. Though in reality the acarus was discovered as early as 1703.

Neither will any one deny that the trichina spiralis is the sole cause of the disease trichiniasis. Still the fact of the trichina having any agency in the causation of disease was not known for 25 years after the discovery of that organism.

The attempt has been made to produce itch by inoculation with the fluid from the vesicles in that disease, but the experiment failed in all cases. The transplantation of the acarus to a new soil, however, always generates itch. The attempt to produce trichiniasis by any other means than through the intervention of the trichina would meet with the same results as would attend the efforts of a hen that, with an ambitious itching for maternity, sought to attain it by setting continuously on chalk eggs.

Any disease which only comes from one particular cause is a specific disease. And, in this sense, typhoid fever, epidemic dysentery and cholera are believed to be as truly specific as syphilis, and the malarial fevers, diphtheria and the exanthematous fevers as much so as gonorrhœa. No amount of decaying matter, no degree of filth can generate a specific disease. No more will the most fertile spot in fruitful Illinois yield a crop of Indian corn without first receiving the seed, than will the foulest and most contaminated locality breed specific diseases without having been sown with the germs of infection. Filth and decaying matter are only a favorable soil for holding and, perhaps, further developing these germs.

It is true, matter undergoing the process of decomposition often produces certain forms of disease; but the symptoms are usually confined to the alimentary canal, and the disease is never communicable. The following incident from the U. S. Government Report of the Cholera Epidemic of 1873, will illustrate the two classes of ailments: Mrs. Brewster, sister to the celebrated Dr. Henry Bennett, having gone to Vienna to attend the Exposition of 1873, put up at a large and commodious hotel, which had just been opened to the public. She complained of the drinking water the first day, and would not even so much as wash in it without first putting in cologne. She, her niece, and maid, and several servants were all attacked with diarrhœa. This was thought to be a bilious complaint, caused by heat, and was looked upon as a trivial matter. Meantime, the water grew worse and worse, and Mrs. B. made complaint to the landlord. She then resorted to milk and mineral water, till the first was found to be diluted with, and the second made from, water on the premises. On the fifteenth day the hot water for the tea at breakfast smelt so badly that the proprietor was sent for, who reported that one of the drainage pipes had broken into his well, but that a dozen men were repairing it. Her niece refused to take the tea, but Mrs. B. had already swallowed some of it. She continued well all day, but about 11 P. M. was attacked with diarrhœa and vomiting, and in six hours was cyanozed and collapsed, and in *fifteen* hours was a corpse.

It was then found that a gentleman had died two days before from cholera in the hotel. Four more died the same day with Mrs. Brewster, while many of the servants and guests were sent to the hospital, where they afterwards died. The water was now examined, and found to be contaminated with sewage. The hotel was at last closed by the police. While the water was simply polluted with ordinary fecal matter, a comparatively trivial diarrhœa prevailed. But so soon as the two cases of true cholera occurred, and the evacuations got into the well, genuine Asiatic cholera at once broke out. This disease had really been in Vienna since the previous April, but the fact was concealed, so as not to compromise the success of the Exposition.

At the siege of Metz it is said that from the bad sanitary conditions then prevailing, many predicted an epidemic of typhus fever. But nothing of the kind occurred, simply because those all-important factors, the seeds of infection, were wanting. Really it seems that whosoever believes in the origin of specific diseases by any other means than through the direct agency of their definite and peculiar causes, is virtually a believer in spontaneous generation, and his faith is not far removed in character from that of those common people who believe that swine, dirt and old shavings will breed fleas.

To my mind, one of the strongest arguments in favor of the germ theory is the striking analogy existing between the growth of plants and the course of certain diseases. Take, for instance, small-pox: Let an unprotected individual be exposed to this disease, and for about twelve days therefrom, which period is called the period of incubation, he will suffer no inconvenience. At the end of this time, however, the disease will make its appearance by the patient having high fever. This continues for a certain time, and is followed by the stage of eruption, which in turn is succeeded by secondary fever, and lastly comes the period of desquamation. Let a grain of corn be put in the ground under suitable conditions, and it first passes through the process of germination, which corresponds very closely to the stage of incubation in small-pox. The next step is vigorous growth for the development of the stalk and leaves; this finds its analogy in the primary fever. Another step is the development of the flower, which, in popular language, is the silk and tassel, and fertilization of the young shoot; this is not unlike the stage of eruption. A fourth step is the growth and development of the ear, which may be compared to the secondary fever. Lastly, we have the drying out and ripening of the ear, which corresponds to the desqumation process in variola.

Here are five stages in either case, and in both instances each stage is within certain limits of definite duration, and corresponds to the carrying out of a certain process. And as we know vegetable life to be at the bottom of all phenomena in the one case, is it unreasonable to suppose that organized action, independent of the body, is the primary cause of what we see taking place in the other. While we are striving to gain light on this subject from analogy, it may not be out of place to give, in a very few words, Lebert's experience with a disease prevailing among silk worms, which was of so fatal a character as almost to destroy the silk industry. He found it to be caused by a minute fungus that infiltrated all the tissues of the body, from the egg to the complete butterfly. This disease had all the characteristics of an epidemic, and was entailed upon the succeeding generation through the agency of the infected eggs.

In the vegetable kingdom each species flourishes best in a certain soil. Some plants require a high, dry location, some do best in a low, wet marsh, others prefer a light, sandy soil, and yet others demand a rich loam. The same is true with infectious diseases. For instance, in small-pox, the disease fastens, by preference, upon the cutaneous system; in typhoid fever, upon Peyer's glands; in dysentery, upon the colon; and in diphtheria, upon the throat; and so on through the whole list.

Ziemssen, in his Cyclopædia of Practical Medicine, makes three classes of infectious diseases. First, those that are directly contagious, as variola, measles, typhus fever, etc. Secondly, those diseases which are purely miasmatic, in which the poison comes from without, never having had previous connection with the body. The malarial diseases belong to this group. The third class is not so simple in its manner of production. To make this plain, a little illustration is necessary.

It is said that an injection of blood from a cholera patient into a healthy subject will not cause the disease. It is also asserted that fresh cholera discharges are incapable of generating cholera; but that these discharges, or rather the germs which they are believed to contain, have to go through a process of further development before they can produce the disease. Contact with decomposing matter is highly conducive to this result.

Here, then, we have a poison which is generated in the body, but which has to be subjected to influences *without* the body before it is capable of reproducing its kind. So that a third group is called the miasmatic-contagious. Cholera, typhoid fever and dysentery are good examples. By the etiological system, typhoid fever, so far from being *like* typhus, does not even belong to the same group; typhoid belongs to the miasmatic-contagious class, while typhus is grouped with the directly contagious maladies. The miasmatic-contagious diseases, in their manner of development, bear a striking resemblance to those plants which have to be first sprouted in hotbeds before they are in a fit condition to be put in their proper soil for further growth.

Liebermeister suggests that the difference between the diseases that are directly contagious and the miasmatic-contagious group is not so great as a superficial examination would lead us to suppose. He surmises that the change through which the germs of cholera, for instance, have to pass outside the body, before they can reproduce that disease, takes place in the body in the case of the contagious diseases during the stage of incubation. Or, in other words, we may regard the body as a kind of soil, in which flourish certain diseases, and in the case of the miasmatic contagious class, the germs are, so to speak, sprouted without the body, and are then received, and at once produce their peculiar results. In the contagious diseases, however, the seeds are, as it were, received directly and sprouted in the same soil in which they are to grow.

Zymotic is a term that has long been applied to infectious diseases; but as zymosis, or the process of fermentation, has been shown to depend upon the presence of vegetable organisms, the zymotic theory of the origin of this class of diseases becomes identical with the germ theory.

